# The Freiburg Acuity Test in Preschool Children: Testability, Test–Retest Variability, and Comparison With LEA Symbols

**DOI:** 10.1167/tvst.13.3.14

**Published:** 2024-03-19

**Authors:** Navid Farassat, Vanessa Jehle, Sven P. Heinrich, Wolf A. Lagrèze, Michael Bach

**Affiliations:** 1 Eye Center, Medical Center–University of Freiburg, Faculty of Medicine, University of Freiburg, Freiburg, Germany

**Keywords:** FrACT, LEA symbols, visual acuity, test–retest variability, Landolt C

## Abstract

**Purpose:**

To determine the testability, performance, and test–retest variability (TRV) of visual acuity (VA) assessment using the Freiburg Visual Acuity Test (FrACT) compared to the LEA Symbols Test (LEA) in preschool children.

**Methods:**

In 134 preschool children aged 3.0 to 6.8 years, monocular VA of each eye was measured twice with a four-orientation Landolt C version of the FrACT and once with the LEA. FrACT runs were preceded by a binocular run for explanatory purposes. Test order alternated between subjects. Optotypes were presented on a computer monitor (FrACT) or on cards (LEA) at a distance of 3 m.

**Results:**

Overall, 68% completed the FrACT (91/134 children) and 88% completed the LEA (118/134 children). Testability depended on age: FrACT, 19% (<4 years) and 87% (≥4 years); LEA, 70% (<4 years) and 95% (≥4 years). Mean ± SD VA difference between tests was 0.11 ± 0.19 logarithm of the minimum angle of resolution [logMAR], with LEA reporting better acuity. The difference depended on age (0.27 ± 0.23 logMAR [<4 years], 0.09 ± 0.18 logMAR [≥4 years], *P* < 0.001) and on test sequence (higher age dependence of FrACT VAs for LEA first, *P* < 0.001). The 95% limits of agreement for the FrACT TRV were ±0.298 logMAR.

**Conclusions:**

The examiner-independent FrACT, using international reference Landolt C optotypes, can be used to assess VA in preschool children aged ≥4 years, with reliability comparable to other pediatric VA tests.

**Translational Relevance:**

Use of the automated FrACT for VA assessment in preschool children may benefit objectivity and validity as it is a computerized test and employs the international reference Landolt C optotype.

## Introduction

Visual acuity (VA) assessment constitutes an essential component in every eye examination, in both children and adults. It has a critical impact on quality of life^1^ and is the most common primary outcome measure in clinical studies. In literate adults, the ETDRS protocol is recognized as a reference for clinical trials,^2^ while the eight-orientation Landolt C is the international standard optotype defined by ISO 8596.^3^ In Germany, the national standard DIN 58220-3, based on the international standard ISO 8596, prescribes the Landolt C optotype for use when VA is tested as part of a medical expert opinion or fitness-to-drive examination.^4^

There are no such standards for examinations in children, although some testing protocols, such as HOTV, have been proposed, particularly by the PEDIG group (Holmes JM 2001).^18^ This lack of international standards is due to the age-dependent heterogeneity in visual function and cognitive skills in children. Quantifying visual acuity in early childhood is challenging due to several factors such as illiteracy, limited communication skill, short attention span, lack of compliance, and high examiner dependency, as well as the variable impact of the crowding phenomenon.^5^^–^^8^ Nevertheless, VA assessment in children is of great importance in screening for amblyopia and other eye diseases.

Accordingly, there are a variety of VA tests for children in different age groups. In infants and toddlers, visual evoked potentials^9^^,^^10^ and optokinetic nystagmus^11^^,^^12^ have been used to objectively measure VA. Other methods used in preverbal children include preferential looking tests such as the Teller Acuity Cards^13^ and the Cardiff Acuity Test.^14^ Verbal yet preliterate children aged 3 years and older can complete recognition acuity tests, which require naming or matching of pictures/symbols or letters as optotypes (e.g., Allen Cards,^15^ Wright Figures,^16^ Kay Pictures,^17^ the HOTV test,^18^^,^^19^ the Sheridan–Gardiner test,^20^ and the LEA Symbols Test^21^). In the same age group, Landolt C and Tumbling E charts are being used as resolution acuity tests.^22^^,^^23^ Older children, who are already familiar with letters, can reliably perform VA test using adult letter charts such as the ETDRS.^24^^–^^26^

For VA tests using differently shaped optotypes (numbers, letters, symbols), there is evidence suggesting that some optotypes are more easily recognizable than others within the same test. This could systematically bias any VA assessment.^27^ In contrast, Landolt C charts are well standardized and designed to offer equivalence between distinct optotypes, differing only in their respective gap positions.

Such standardized Landolt C optotypes are used in the Freiburg Visual Acuity Test (FrACT), a computer-based, examiner-independent VA test battery complying with ISO 8596. This test has been developed and described in detail by one of the authors,^28^^–^^30^ runs on multiple operating systems, can be downloaded free of charge, and has been validated in various studies.^31^^–^^33^ As VA testing in children is known to be highly examiner dependent, it could benefit from the examiner-independent nature of FrACT.

Therefore, the primary purpose of this study was to investigate testability, performance, and test–retest variability (TRV) of the FrACT as an examiner-independent, automated VA test employing standardized Landolt C optotypes. Another important purpose of this study was to compare the testability and performance of the FrACT with that of a widely used clinical pediatric VA test, the LEA Symbols Test, as routinely used in our pediatric clinic and as recommended in the German guideline concerning amblyopia.^34^

## Materials and Methods

### Study Participants

For this observational, cross-sectional study, German preschool children were recruited from the outpatient clinic of the Eye Center of the University of Freiburg Medical Center and from two childcare institutions in the region between March and October 2011.

Inclusion criteria were as follows:

• Age from 3 to 6 years
• Informed consent of the respective legal guardians

Exclusion criteria were a clinical diagnosis of either

• Mental retardation
• Abnormal age-related development (fine/gross motor skills, speech, cognitive skills).

In addition, 19 adolescents and adults (38 eyes) with normal ophthalmologic status aged 14 to 55 years were recruited.

### Ethics

Ethics committee approval was obtained (Ethics Committee, University of Freiburg, #366/10). We complied with the Declaration of Helsinki, local laws, and International Council for Harmonisation of Technical Requirements for Pharmaceuticals for Human Use – Good Clinical Practice (ICH-GCP).

### VA Testing Protocols

All participants underwent monocular VA testing of each eye with two *methods*: the FrACT and the LEA Symbols Test (LEA). All tests were performed in an artificially lit room by a single experienced examiner (VJ). The participant was placed alone or on a parent's lap 3 m from the display or LEA chart, respectively (see below). There was no feedback indicating correctness of the responses. The *sequence* (FrACT or LEA first, no randomization) was alternated between participants. The right eye was always tested first. The other eye was occluded with an adhesive patch. Care was taken to ensure that the study conditions at the Eye Center of the University Medical Center Freiburg and at the childcare institutions were similar. For example, the same computer and display were used for all computer-based testing. The viewing distance (3 m), display brightness (180 cd/m², measured with a Minolta Spotmeter F; Konica Minolta, Osaka), and ambient illuminance (higher than 1% of the display brightness but lower than the display brightness, in accordance with https://michaelbach.de/fract/checklist.html) were kept constant. The dimensions of the rooms where VA testing took place were similar. All VA tests were performed in compliance with ISO 8596.

### FrACT

The FrACT is a standardized, validated, computerized visual test developed and described in detail by one of the authors.^28^^,^^29^^,^^35^ In short, FrACT tests VA following the Best PEST (parameter estimation by sequential testing) algorithm.^36^ Here, the VA threshold estimation was performed as previously described.^28^^,^^29^ The Best PEST algorithm calculates the inflection point of the constant fixed slope of the psychometric function of VA. Due to a guessing rate of 25% in our study (four optotypes), the inflection point is where the guessing probability (*G*) equals 62.5%:

$$\begin{array}{c} G=0.5\left(1-0.25\right)+0.25=0.625. \end{array}$$

After each trial (Landolt C presentation and participant response), this algorithm calculates the most likely VA based on all previous trials. The corresponding Landolt C size is chosen for the next stimulus presentation. Step sizes take the logarithmic nature of perception into account since the Best PEST algorithm operates on a log(arcmin) scale. Initially, step sizes are quite large (∼3 VA lines) but become smaller the more information on the threshold becomes available via the responses. This results in a smaller number of optotype presentations far away from the patient's VA and consequently a higher number of optotype presentations close to the patient's VA.

In this study, the FrACT was performed at a distance of 3 m, first binocularly as a practice run and then monocularly, twice per eye (in the order OD, OS, OD, OS). With the given monitor size and resolution, VAs from 2.0 to −0.3 logarithm of the minimum angle of resolution (logMAR) could be tested at a distance of 3 m. To increase comprehensibility for children, only four different Landolt C orientations (up, right, down, left) were presented instead of the eight orientations normally used.

Individual standard Landolt C optotypes were displayed on a computer screen as optotypes in black color on a white background. The FrACT uses antialiasing to improve spatial resolution of computer displays in order to assess VA with high resolution without increasing the viewing distance.

Thirty Landolt C rings were shown per run, making a total of 150 Landolt C rings per participant (one binocular test run and two monocular runs per eye). Depending on the child’s preference, a suitable story was invented to promote concentration during the test (e.g., “where can the mouse escape?”). Small breaks (<2 minutes) were taken after each run (i.e., after 30 Landolt Cs). More frequent breaks were given when needed. The children entered the responses via a keypad, where the buttons were spatially arranged in correspondence with the four gap directions of Landolt C rings (e.g., Landolt C with the gap at the top corresponded to the button on the remote control being at the top).

### LEA

The LEA is a symbol optotype test (four symbols: circle, heart/apple, square, house) commonly used in preliterate children. The LEA Symbols are based on the same principles as the Bailey–Lovie chart^37^ and were developed by Lea Hyvärinen et al.^21^ for better standardization: on each line, there is the same number of optotypes, whose sizes decrease exponentially from line to line. On average, the symbol sizes are 1.5 times larger than the corresponding Snellen E optotypes, so that adult participants achieve the same level of VA. Overall, children show good cooperation on the LEA Symbols test, especially children 3 years and older.^38^^–^^40^

In this study, the LEA test was performed monocularly, once per eye, at a distance of 3 m. We used uncrowded LEA Symbols VA charts (Lighthouse Single Symbol Book, #250600; LEA Test International, LLC, Etters, Pennsylvania, USA). The children’s responses were either naming or matching the symbols, depending on the child's preference. The other symbols on the respective page of the book were covered so that only one symbol was visible at a time. Each VA level contains four symbols/optotypes for each decimal logarithmic step from 2.0 to −0.3 logMAR (24 VA levels total) calibrated to a 3-m test distance. VA was determined using a three-out-of-four criterion following a four-alternative forced-choice procedure. The highest VA at which the participant was able to identify at least three of four optotypes was recorded as the VA. There was 1 trial (= 4 symbols) at each acuity level and therefore a maximum number of 24 trials (= 96 symbols). Trials for individual test levels were not retested.

### Statistics

Analysis was carried out using the statistical analysis package R.^41^ Statistical analyses of VA were performed on the nearly normally distributed log(VA_decimal_) = −logMAR scale. A probability value of <0.05 was considered statistically significant. Data values are presented as means ± SDs.

#### Testability

If the VA test of any eye could not be completed to the last symbol (i.e., 30 of 30 optotypes for the FrACT), that participant’s VA assessment was considered not testable (i.e., successful testability implies successful testing with each eye). Similarly, for participants who could only complete one of the two VA tests (FrACT or LEA), the other test was considered unsuccessful. For the FrACT to be considered testable, only one of the two FrACT runs for an eye had to be successfully completed with each eye. However, there were no cases where one FrACT run was successful and the other was not.

To obtain a measure of dispersion for the testability, 2.5% to 97.5% confidence intervals were calculated in R via bootstrapping, using the “resample” method from the “modelr” package. The results are based on 10,000 samples.

Significance was tested using the Mann–Whitney *U* test.

#### VA Depending on Age, Sex, and Method

A mixed analysis of variance (ANOVA) was set up with age and sex as between factors and method (LEA or FrACT) as a within factor using the “aov” procedure of R.

#### VA Differences Between FrACT and LEA

Only the first FrACT run was taken for comparison with the LEA test. To plot VA differences measured with the comparable LEA and FrACT as a function of age, linear models were fit using the “lm” function of R.

#### Test–Retest Variability

This could only be tested in the FrACT with its two runs and was quantified via the 95% limits of agreement (LoA)^42^ analyzed by comparing both test runs performed with each eye. LoA was calculated as follows:

$$\begin{array}{c} \mathrm{LoA}=1.96\cdot \mathrm{sd}\left(\mathrm{test}2i-\mathrm{test}1i\right) \end{array}$$

where

*SDi* = standard deviation of the difference between the first and second test runs per participant.

## Results

### Study Population

**Figure 1.**
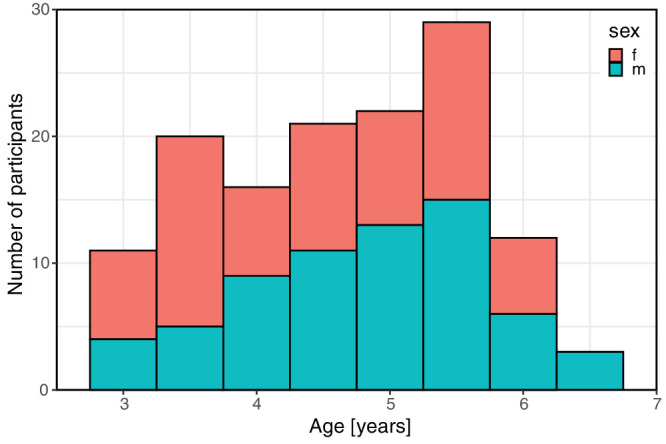
Age and sex distribution of study population. Female participants are shown in *red*, male participants in *blue*. Data at age label “3” comprises the interval [3; 3.5) years of age and so on.

We included a total of 134 children in this study, 76 from the outpatient clinic of the Eye Center of the University Medical Center Freiburg and 58 children from two childcare institutions. Sixty-six children (49%) were male and 68 (51%) female. The children were 3.0 to 6.8 years old (mean age, 4.7 years), with most being between 3.0 and 6.0 years old. Figure 1 displays the age and sex distribution of study participants. Twenty-nine children (22%) had a refractive error corrected with spectacles. Of the 134 participants, 61 (46%) had a preexisting eye disease (see Table). Fifteen (11%) of these conditions were relevant to visual acuity, 11 (8%) of which were related to amblyopia.

**Table.** Study Population—Ophthalmologic Diseases

Ocular Disease	Number of Patients
Strabismus without amblyopia	29
Strabismus with amblyopia	7
Anisometropia with amblyopia	3
Chalazion	3
Aphakia	2
Duane syndrome	2
Ptosis without amblyopia	2
Ptosis with amblyopia	1
Aniridia	1
Congenital ocular melanocytosis	1
History of optic papillitis	1
Purulent conjunctivitis	1
Morbus Best	1
Color vision deficiency	1
Corneal scar with mild amblyopia	1
Alternate day squint	1
Megalopapilla	1
Chronic blepharoconjunctivitis	1
Phthisis bulbi after perforating ocular injury	1
Congenital hyperplasia of the pigment epithelium and epiretinal membrane	1

Preexisting ophthalmologic diseases of participants.

### Testability

**Figure 2.**
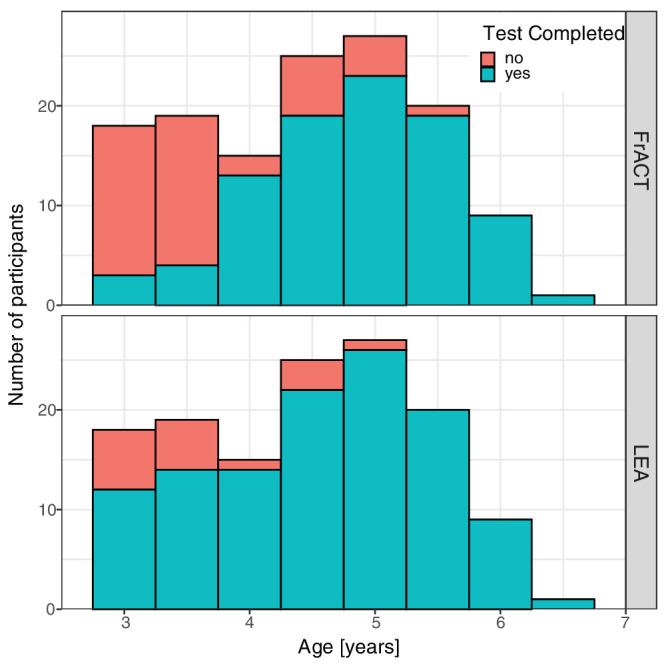
Testability of VA with FrACT (*top*) and LEA (*bottom*). For each age group (in 0.5-year steps), the number of patients with successful (*blue-green*) and failed (*red*) VA test completion is shown. Successful test completion (“yes”) means test completion in each eye. Age ranges as in Figure 1.

Figure 2 shows the testability of the methods depending on the age of the study participants. Overall, 118 of 134 children (88% [82–93%] [actual result and bootstrapped 95% confidence interval]) were able to complete the LEA and 91 of 134 children (68% [60–75%]) were able to complete the FrACT (*P* < .0001).

Testability depended on age: the LEA was testable in most participants, including those aged <4 years (<4 years: 70% [54%–84%]; ≥4 years: 95% [90%–99%]). While few participants under 4 years of age were able to complete the FrACT (19% [8%–32%]), 87% [79%–93%] of children aged 4 years and above completed the FrACT successfully. Testability differed significantly between LEA and FrACT below 4.7 years of age (*P* = 0.033) but not above. Testability was similar in the children examined in the Eye Center of the University Medical Center Freiburg and in the two childcare institutions (segregated data not shown).

### Visual Acuity Depending on Age, Sex, Method, and Sequence

**Figure 3.**
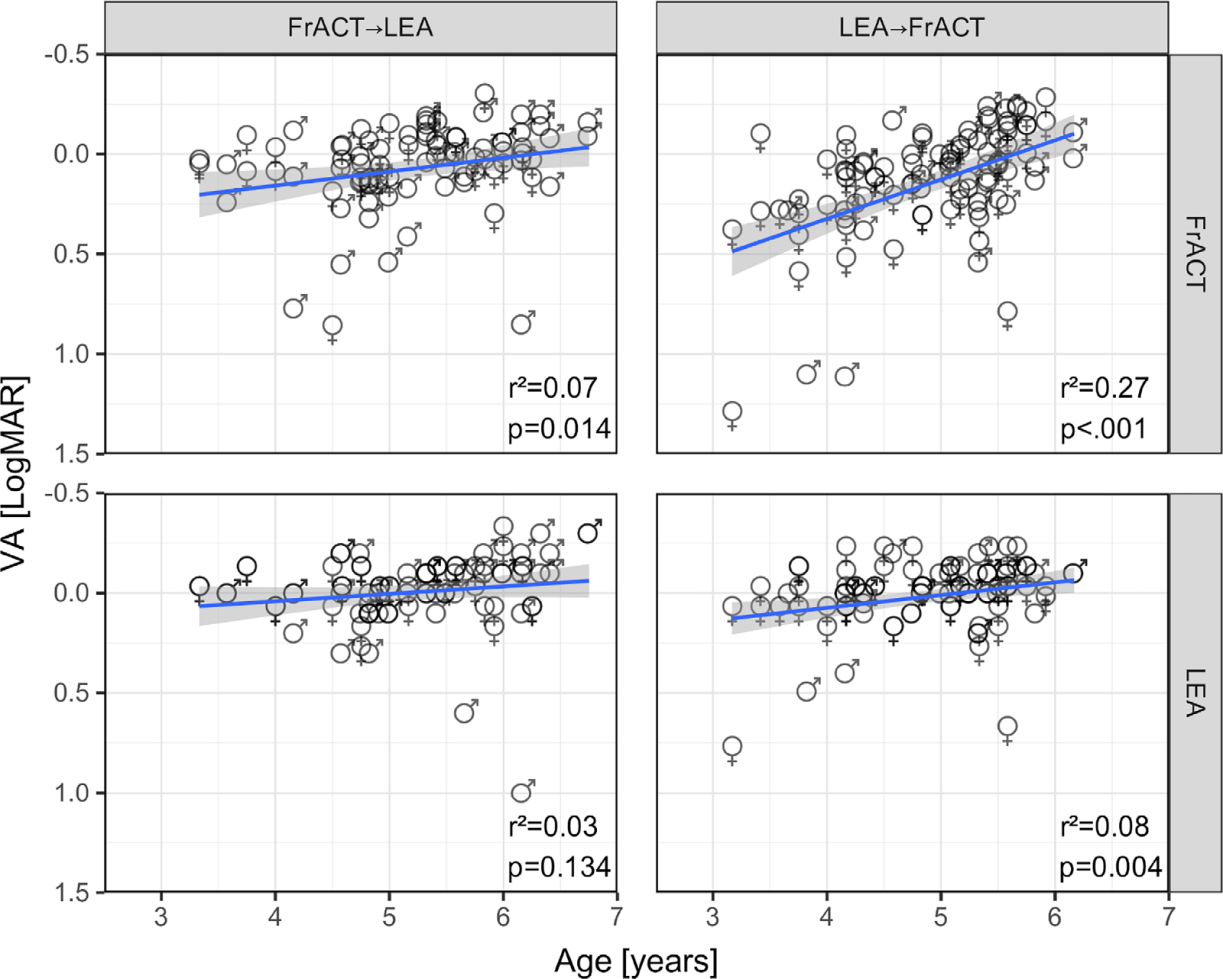
Visual acuity depending on age, sex, method and sequence. Note the inverted logMAR scale: better acuity up. The *straight lines* represent a linear regression, and the *gray shaded areas* surrounding the regression lines indicate the 95% confidence intervals. Individual eyes of female and male participants are shown as corresponding female/male symbols. Only eyes of those participants who successfully completed both tests are included. *Top row*: FrACT VAs. *Bottom row*: LEA VAs. *Left column*: FrACT first. *Right column*: LEA first. *P* values and *r*² are given at the *bottom right* of each panel. Linear regression equations:
FrACT (FrACT→LEA): VA = −0.07 * age + 0.44, FrACT (LEA→FrACT): VA = −0.20 * age + 1.11LEA (FrACT→LEA): VA = −0.04 * age + 0.19, LEA (LEA→FrACT): VA = −0.06 * age + 0.33.

The ANOVA reported significant effects of age (*P* < 0.0001) and method (*P* < 0.0001) but no effects of sex (*P* = 0.37) and sequence (FrACT first or LEA first, *P* = 0.42). There was also a significant interaction of method × age (*P* < 0.0001) and age × method × sequence (*P* = 0.0039). These findings can be graphically appreciated in Figure 3, which plots VA versus age, segregated by method and sequence. As expected, VA improved with age. This age effect differed significantly between methods: the FrACT showed a more pronounced age dependence with a slope of –0.14 logMAR/year (*P* < 0.0001) compared to the LEA with –0.05 logMAR/year (*P* = 0.0017). The higher age dependence using the FrACT was due to a sequence effect with higher age dependence in LEA first compared to FrACT first (Fig. 3, compare top left and top right panels).

### Difference in VA Results Between FrACT and LEA

**Figure 4.**
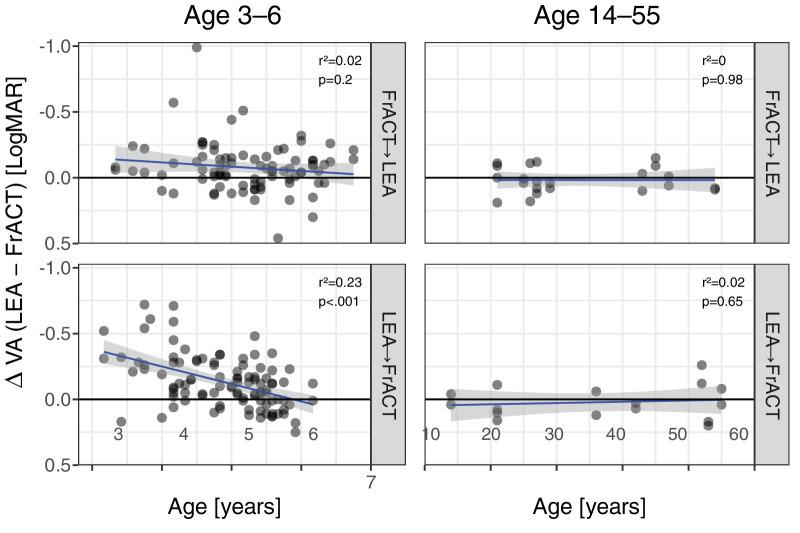
Difference of VA as reported by LEA minus FrACT versus age. Note the inverted logMAR scale: better acuity up. The *thick blue lines* represent linear fits with corresponding 95% confidence intervals (*gray* *shaded*). The ordinate displays the difference between LEA and FrACT VAs (in logMAR) for eyes of participants who completed both tests. On the abscissa, the age range (in years) was divided in children (*left*) and older study participants (*right*). *Top row*: FrACT first. *Bottom row*: LEA first. Note that VA differences in children highly depended on test sequence (compare *left row top* and *bottom*). *P* values and *r*² are given at the *top right* of each panel.

The difference in VA between the FrACT and LEA tests as a function of age and segregated by test sequence is depicted in Figure 4 for the children and an additional control group of 19 older participants (14 to 55 years, mean age: 34.7 years). Whereas the FrACT and LEA tests reported the same mean VA in the older control group regardless of test sequence (mean ± SD; overall: 0.02 ± 0.11 logMAR; FrACT first: 0.02 ± 0.10 logMAR; LEA first: 0.02 ± 0.13 logMAR; *P* = 0.62), there was an age-dependent trend for LEA to report better VAs in preschool children (overall: 0.11 ± 0.19 logMAR; children aged <4 years: 0.27 ± 0.23 logMAR; children aged ≥4 years: 0.09 ± 0.18 logMAR; *P* < 0.001). These age-dependent differences were highly dependent on test sequence: for FrACT first, the differences were not significantly age dependent (overall: 0.08 ± 0.19 logMAR; children aged <4 years: 0.12 ± 0.09 logMAR; children aged ≥4 years: 0.07 ± 0.19 logMAR; see top left panel; *P* = 0.20), whereas LEA first yielded highly age-dependent VA differences (overall: 0.13 ± 0.20 logMAR; children aged <4 years: 0.35 ± 0.24 logMAR; children aged ≥4 years: 0.11 ± 0.18 logMAR; see bottom left panel; *P* < 0.001). The worse VA values reported by FrACT in LEA first (Fig. 3) explain the larger VA differences in LEA first in Figure 4.

### Test–Retest LoA of the FrACT Results

**Figure 5.**
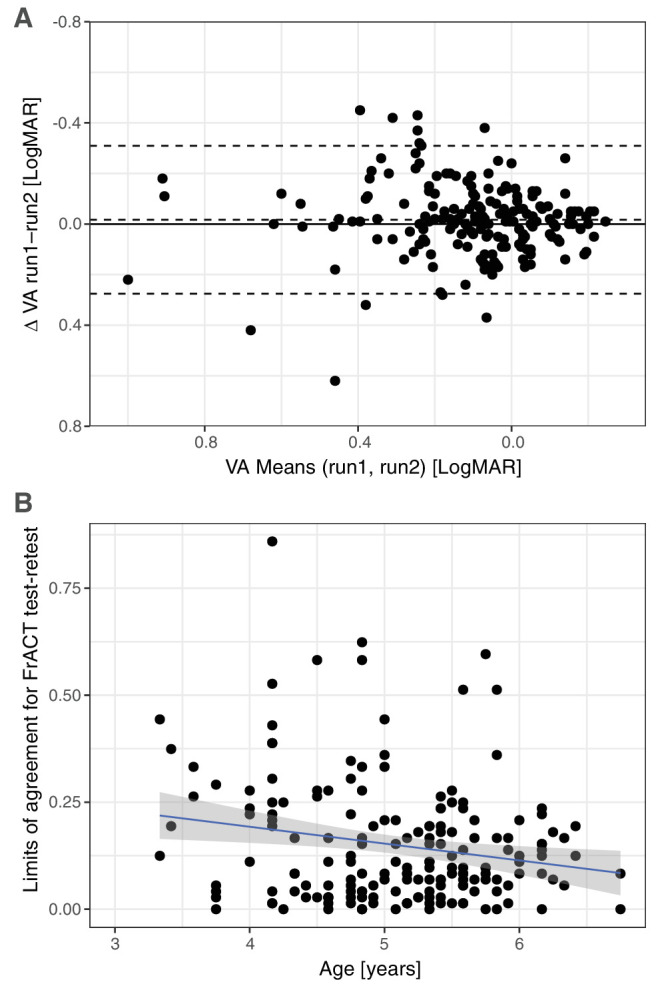
Test–retest variability of FrACT. (**A**) Bland–Altman plot of FrACT results when two runs were available. The *solid line* depicts the *zero line*. Bias (difference between *zero line* and *middle dashed line*) is low (−0.02 logMAR). The *dashed lines* represent ±95% limits of agreement (±0.298 logMAR). (**B**) Individual limits of agreement versus age, showing wide variability and a small decline with age (*r* = −0.20, *P* = 0.0075; LoA = 0.35–0.039 * age): the linear regression explains only 4% of the variance.

Test–retest properties were quantified via LoA^43^ and a Bland–Altman plot (Fig. 5A). We found a negligible bias (0.017 logMAR, better VA for the first test run) and a range of ±0.298 logMAR for the 95% LoAs of TRV (average of all preschool children). Age-specific LoAs were ±0.363 logMAR below 4 years of age and ±0.289 logMAR above. The age dependence of the TRV shows a wide distribution; its slight decrease with age (Fig. 5B, linear regression, *r* = −0.20, *P* = 0.0075; LoA = 0.35–0.039 * age) only explains 4% of the variability. In the control population of 19 adults and adolescents, the 95% LoAs of TRV were ±0.154 logMAR.

## Discussion

### Differences in Testability Between FrACT and LEA

Testability was age and test dependent. FrACT, for which use in very young preschool children has not been reported previously, showed high testability from the age of 4 years. LEA, which is already widely adopted for use in preschool children,^8^ was possible in all examined age groups (3–6 years). This agrees with and extends examinations in a German cohort of preschool children (340 children with an average age of 5.1 years) at school enrollment^22^: in that study, VA assessment using the FrACT and Tumbling E chart was compared. Testability of both FrACT and Tumbling E was 99%, and agreement between FrACT and Tumbling E was very high (approximately ±1 line).

In the present study, completion rates of FrACT and LEA in older preschool children (≥4 years of age) were above 85% (FrACT, 87%; LEA, 95%). Younger preschool children (<4 years of age) showed lower testability with the FrACT (FrACT, 19%; LEA, 70%); the difference in testability was significant below 4.7 years of age. This may reflect the influence of human interaction in VA assessment, particularly at a very young age: Children's responses on the LEA test could be positively biased by human interaction. In contrast, the lack of human interaction in the automated FrACT may lead to reduced compliance.

### Visual Acuity Depending on Age and Method

The ANOVA of the VA results revealed a number of expected and unexpected findings: as previously reported,^8^ we found better results in older children. Besides this, we found that the LEA test yielded better overall VA results, which is in agreement with previous reports.^38^^,^^44^^,^^45^ In those studies, VAs measured with Landolt C charts were 0.07 to 0.14 logMAR worse compared to those measured with LEA Symbols—in both children and adults. Better acuity for LEA was also found in comparison to the Tumbling E chart,^46^ the Bailey–Lovie Letter chart,^47^ the Patti Pics chart,^48^ and the ETDRS chart.^49^ Thus, VA scores achieved in LEA appear to be consistently better than those achieved in other VA tests, especially for the youngest participants. It has been proposed that this discrepancy is due to the fact that LEA Symbols and Landolt rings measure different visual acuity components. In contrast to Landolt rings, LEA Symbols do not differ from each other in only one detail but contain complex spatial information. Thus, while Landolt C rings are supposed to determine the minimum separable or resolution acuity, LEA Symbols actually measure shape recognition acuity, the so-called minimum cognoscible.^8^^,^^44^ This is of importance, since resolution acuity depends more on the quality of the retinal image, whereas recognition acuity demands more cognitive skills. However, the notion that Landolt C optotypes determine resolution acuity has been disputed.^6^^,^^50^

Other possible explanations for the VA differences between LEA and FrACT reported in this study are (1) the high number optotype presentations with the FrACT (see below) and (2) the additional psychomotor hurdle of matching Landolt C directions and pressing the correct button on the remote control in the FrACT.

### Effect of Sequence

The ANOVA revealed a highly significant interaction of the factors age, method, and sequence. As illustrated in Figure 3, FrACT VAs showed the greatest age dependence when the LEA test was performed first (i.e., worse VAs in younger children using the FrACT when the LEA test was performed before). We hypothesize that the high number of optotype presentations (FrACT: 5 times 30 optotype presentations [one binocular test run and two monocular runs per eye]; LEA: maximum number of 24 trials per eye [from 2.0 to −0.3 logMAR in steps of 0.1 logMAR] with four optotype presentations each = maximum 192 optotype presentations in total) was too demanding, especially for the youngest children, indicating that our study may overestimate VA differences between LEA and FrACT in children <4 years of age. This also suggests that the FrACT TRVs are possibly overestimated and would improve with a smaller number of trials. Indeed, in a more recent work,^22^ we found 18 instead of 30 trials for the FrACT to be sufficient.

### TRV

In the present study, we determined the TRV of VAs in preschool children and in an older control group using the FrACT (but not the LEA as it was only tested once). The mean TRV of FrACT VAs (given as the 95% LoAs) in preschool children was about ±0.3 logMAR (with some age dependence: ±0.363 logMAR in children <4 years of age, ±0.289 logMAR in children ≥4 years of age). The high TRV found in our study is similar to those reported for other VA testing methods in children: using Landolt C charts in school children aged 6 to 9 years, Schmidt-Bacher et al.^51^ measured TRVs of ±0.24 to ±0.32 logMAR. Chen et al.^52^ reported a TRV of ±0.18 logMAR for LEA in amblyopic and healthy children aged 4 to 12 years. Shah et al.^53^ found TRVs of ±0.14 to ±0.16 logMAR in amblyopic children aged 4 to 15 years, employing printed or computerized crowded Kay Pictures and ETDRS. Others have found similar values.^18^^,^^54^^–^^56^ Possible reasons for the high TRV in our study compared to others include differences in testing protocols (test method, mode of optotype presentation mode of response, and as described above especially the high number of optotype presentations) and differences in study populations (younger children in our study).

In adults, TRVs are smaller, ranging from about ±0.1 to ±0.2 logMAR using different VA charts.^57^^–^^64^ The TRV of VAs of adults using the FrACT has been reported as ±0.2 logMAR.^29^ In the present study, the TRV of FrACT VAs in a control group of 19 adults and adolescents has been ±0.154 logMAR.

### Limitations

Our study has some limitations: first, the study design makes a direct comparison between Landolt C optotypes and LEA symbol optotypes difficult, as the testing protocols differed between the FrACT and the LEA: Landolt C optotypes as part of the FrACT were presented on a computer screen and children responded by remote control, whereas LEA Symbols were offered on cards and children responded by naming or matching the recognized symbol on a board. As explained above, the number of optotype presentations in the FrACT was very high. A reduction in the number of presentations might have increased testability without undue loss in precision, especially in younger children. Also, the FrACT and the LEA test differed in their threshold criteria, with the FrACT set at 62.5% guessing probability calculated by the Best PEST algorithm and the LEA at 75% by a three-out-of-four forced-choice procedure. We did not correct for the difference between thresholds. In addition, although care was taken to ensure that the study conditions at the Eye Centre of the University Medical Centre Freiburg and the childcare centers were similar, we cannot exclude a bias due to small differences in setting, for instance, lighting and distractors. Also, the use of a single experienced examiner could have introduced bias toward one test method. Finally, we did not analyze whether LEA or FrACT performs better in children with poor VA since our sample size of such children was too small.

## Conclusions

In conclusion, the FrACT, as an examiner-independent tool using the international reference Landolt C optotype, can be used to assess VA in preschool children aged 4 years and older, with reliability comparable to other assessment methods reported in the literature. In this age group, testability (87%) and TRV (±0.29 logMAR) support the use of the FrACT for VA assessment in preschool children. However, the FrACT has limited utility in children under 4 years of age, where the LEA test showed higher testability and a three-line better VA. Given the limitations of our study concerning design and methodology (e.g., number of optotype presentations), further investigations are needed to validate the FrACT against pediatric VA tests and in children with visual impairment.
